# *SF3B1* mutation is a poor prognostic indicator in luminal B and progesterone receptor-negative breast cancer patients

**DOI:** 10.18632/oncotarget.22983

**Published:** 2017-12-05

**Authors:** Xing Fu, Ming Tian, Jia Gu, Teng Cheng, Ding Ma, Ling Feng, Xing Xin

**Affiliations:** ^1^ Department of Pediatric Hematology, Children’s Hospital of Hainan Province, Haikou 570000, Hainan, P.R.China; ^2^ Department of Nephrology, Puai Hospital, Tongji Medical College, Huazhong University of Science and Technology, Wuhan 430030, Hubei, P.R.China; ^3^ Department of Hematology, Tongji Hospital, Tongji Medical College, Huazhong University of Science and Technology, Wuhan 430030, Hubei, P.R. China; ^4^ Department of Breast and Thyroid Surgery, Tongji Hospital, Tongji Medical College, Huazhong University of Science and Technology, Wuhan 430030, Hubei, P.R.China; ^5^ Department of Obstetrics and Gynecology, Tongji Hospital, Tongji Medical College, Huazhong University of Science and Technology, Wuhan 430030, Hubei, P.R.China

**Keywords:** SF3B1 mutation, luminal B, progesterone receptor-negative (PR-negative), breast cancer, prognostic parameters

## Abstract

The purpose of this study was to explore the relationship between SF3B1 mutations and the prognoses of patients with breast cancer. Clinical and SF3B1 mutation data from The Cancer Genome Atlas were analyzed. SF3B1 mutations were evaluated as prognostic factors in all breast cancer patients and specific subgroups through Cox regression and Kaplan-Meier analyses. We also investigated the relationship between traditional parameters and SF3B1 mutations. Receiver operating characteristics curves were used to analyze common risk factors for their sensitivity and specificity in predicting SF3B1 mutations. SF3B1 mutations were a poor prognostic factor in luminal B and progesterone receptor (PR)-negative breast cancer (*P* < 0.01). Age at diagnosis and estrogen receptor (ER) status were associated with SF3B1 mutations in all breast cancers (*P* < 0.01) and in luminal B and PR-negative subgroups (*P* < 0.01). The age at diagnosis and ER status combined had a higher sensitivity and specificity for predicting SF3B1 mutations than each factor alone. SF3B1 mutations are a poor prognostic factor in luminal B and PR-negative breast cancer patients. These mutations are significantly associated with age at diagnosis and ER status. SF3B1 mutations may therefore be a novel therapeutic target for breast cancer patients.

## INTRODUCTION

The precise excision of introns from precursor mRNAs in eukaryotes is performed by the spliceosome [1], a macromolecule composed of small nuclear RNAs associated with proteins [2]. RNA splicing, which includes constitutive and alternative splicing, is a post-transcriptional process necessary to produce mature RNA [3]. Constitutive splicing is the process of removing introns from pre-mRNA, whereas alternative splicing is the process of including or excluding exons in different combinations to create a diverse array of mRNA transcripts from a single pre-mRNA fragment. SF3b is a heptameric protein complex of the U2 small nuclear ribonucleoprotein that is essential for pre-mRNA splicing. Mutations in the largest SF3b subunit, SF3B1/SF3B155, are linked to cancer and lead to alternative branch site selection [4, 5]. The *SF3B1* gene encodes subunit 1 of the splicing factor 3b, which is important for anchoring the spliceosome to the precursor mRNA [2], and is the most commonly mutated gene found in myelodysplastic syndrome [6]. The frequency of *SF3B1* mutations is particularly high among the unique subtypes of myelodysplastic syndrome that are characterized by increased ring sideroblasts, in which mutation frequencies of 66.7–79% have been reported [2, 7, 8]. *SF3B1* knockout mice are embryonic lethal at very early developmental stages, whereas *SF3B1* heterozygous knockout mice (*SF3B1*^+/−^) exhibit mild skeletal alterations [9]. *SF3B1* was found to be the second most frequently mutated gene in chronic lymphocytic leukemia (CLL) at 5–15%; *SF3B1* mutations are less common in the early stages of CLL and become more prevalent in advanced disease where they tend to be associated with poor prognosis. The K700E mutation accounts for more than 50% of the variants observed, and additional codons 666, 662, and 625 were found to be hot spots for mutation [2, 10]. In addition to hematological malignancies, lower frequencies of *SF3B1* mutation are also found in solid tumors such as breast cancers (1.8%), pancreatic carcinoma (4%), uveal melanoma (9.7%), and endometrial cancers (percentages not reported) [9]. Patients with uveal melanoma who harbor *SF3B1* mutations are reported to have better prognoses [11, 12].

Human breast cancers are heterogeneous, and patients have varying clinical outcomes based on their diagnostic and prognostic parameters. These include morphological assessment, basal-like phenotype, and the expression statuses of estrogen receptor (ER), human epidermal growth factor receptor 2 (HER2), and progesterone receptor (PR). [13–15]. The genomic landscape of breast cancer is complex, and somatic mutations related to this disease have been extensively characterized.

RNA splicing dysfunction may be associated with the pathogenesis of breast cancer, as Maguire et al. revealed that mutations in spliceosomal component genes occur in 5.6% of breast cancers. Mutation of *SF3B1* in spliceosomal component genes was the most common in breast cancers, and was detected in approximately 1.8% of cases. *SF3B1* hotspot mutation K700E was detected in 16% and 6% of papillary and mucinous breast cancers, respectively [11]. These *SF3B1* K700E mutations could lead to differential splicing. Alternative splicing of genes has also been shown to be associated with *SF3B1* mutations in breast cancer, such as *TMEM14C*, *RPL31*, *CRNDE*, *DYNLL1*, *MZB1*, *ICA1*, *RPL24*, *MTERFD3*, *OBSL1*, *ABCC5*, *UQCC*, *GUSBP11*, *ANKHD1*, *ADAM12*, *F8*, and *GAS8* [11, 12]. Pereira et al. also showed that recurrent K700E mutations in *SF3B1* are associated with differential splicing activity in breast cancer; they found that patients presenting with mutations in *SF3B1* tended to be older [14]. We performed this study to comprehensively investigate the association between *SF3B1* mutations and prognoses in breast cancer patients.

## RESULTS

### Frequency of *SF3B1* mutation in carcinoma

**Figure 1:**
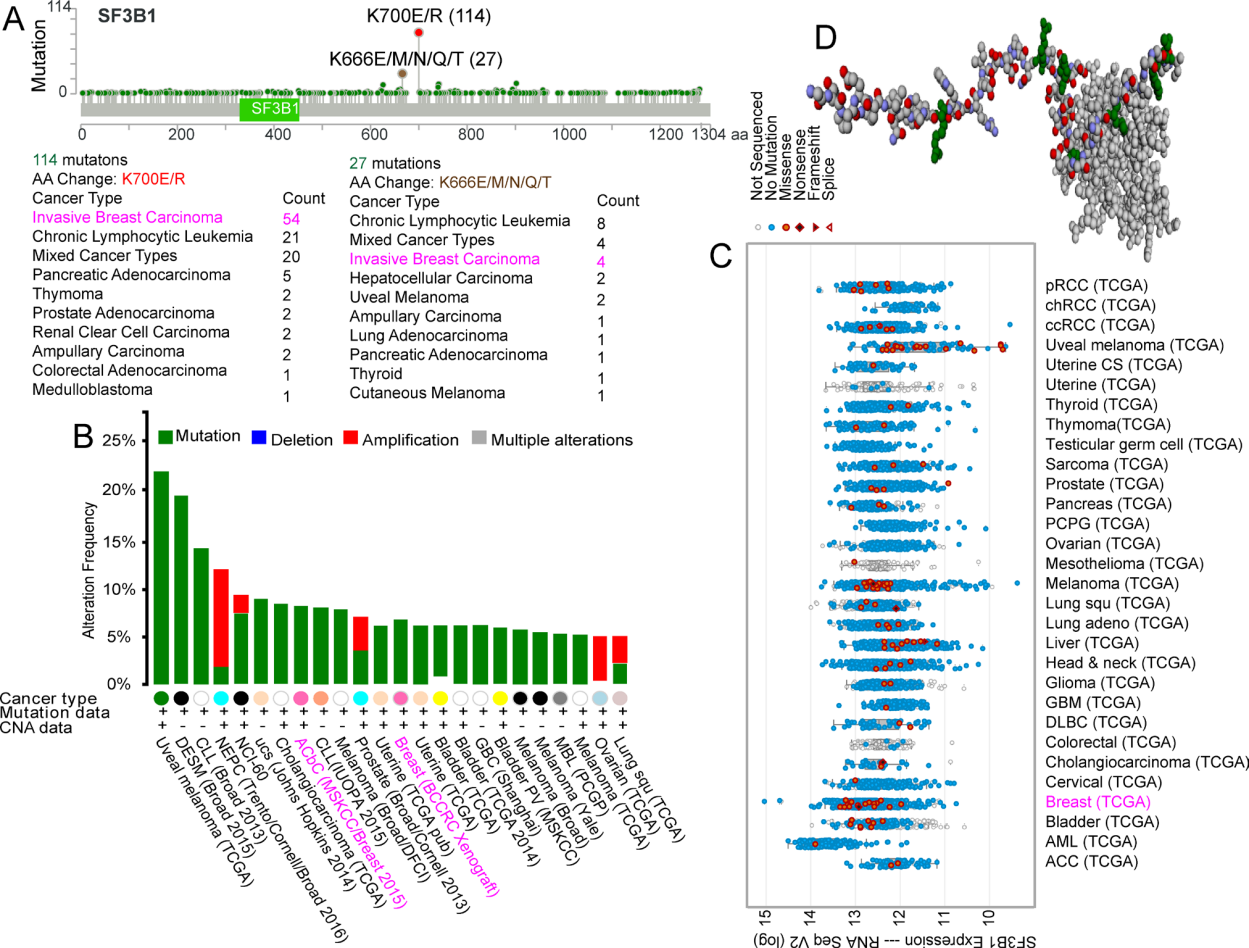
Frequency of *SF3B1* mutations in carcinoma *SF3B1* K700E/R and K666E/M/N/Q/T hotspot mutations (**A**), *SF3B1* mutations frequency (**B**), and *SF3B1* expression with mutations (**C**) in different types of carcinoma. Shown is the structure of the SF3b155 peptide complex derived from the cBioPortal for Cancer Genomics (**D**). [1, 4].

Analysis of the cBioPortal for Cancer Genomics (www.cbioportal.org/) revealed 114 *SF3B1* K700E/R hotspot mutations across different types of carcinoma, 54 of which were in invasive breast carcinoma. Four patients with invasive breast carcinoma among 27 with various carcinoma types carried the *SF3B1* K666E/M/N/Q/T hotspot mutation (Figure 1A). In terms of mutation frequency among different carcinomas, we found that the *SF3B1* mutation ranged between 5% and 10% in breast cancer (Figure 1B). *SF3B1* expression was demonstrated in different TCGA carcinoma study groups (Figure 1C). The spliceosomal protein SF3b155 structure is shown in Figure 1D [1, 4].

### Clinical characteristic and prognostic factors

The median age of breast cancer patients at diagnosis was 60.09 years (range, 22–96 years). There were 2061 and 1221 patients < 65 years and ≥ 65 years, respectively. The Nottingham prognostic index (N-Index) was only found in the METABRIC data sets. The N-Index was < 4.05 in 1075 patients and ≥ 4.05 in 910 patients. Eighty-one of 3817 patients (2.12%) carried *SF3B1* mutations. The clinical characteristics of the breast cancer patients are shown in Table 1. Cox univariate analysis showed that the prognostic factors significantly associated with overall survival (OS) were age, N-Index, ER status, PR status, HER2 status, menopausal status, PAM-50 and claudin-low subtype, neoplasm histologic grade, breast cancer type, and tumor stage. However, *SF3B1* mutations and breast cancer laterality were not associated with OS.

**Table 1:** Clinical characteristics of breast cancer patients and their correlation with overall survival

		patients (*n*)	percentage (n/N%)	HR 95% CI	*P*
Age (years)					
	< 65	2061	54%		
	≥ 65	1221	32%	2.113 (1.893, 2.360)	0.000
	Lost	535			
N-index					
	< 4.05	1070	28%		
	≥ 4.05	910	23%	1.957 (1.741, 2.200)	0.000
	Lost	1837			
SF3B1 mutation					
	Yes	81	2.10%		
	No	3736	97.90%	1.154 (0.835, 1.593)	0.385
ER status					
	Positive	2438	63.90%		
	Negative	762	20%	0.848 (0.746, 0.965)	0.012
	Lost	617			
PR status					
	Positive	1844	48.30%		
	Negative	1357	35.60%	0.784 (0.703, 0.875)	0.000
	Lost	616			
HER2 status					
	Positive	464	12.20%		
	Negative	2375	62.20%	1.420 (1.216, 1.659)	0.000
	Lost	978			
Menopausal state					
	Pre	681	17.80%		
	Post	2280	59.60%	1.734 (1.487, 2.022)	0.000
	Lost	856			
PAM50 and claudin-low subtype					
	Normal	157	4.10%		
	luminal A	736	19.30%		
	Luminal B	497	13%		
	Claudin-low	218	5.70%		
	HER2	242	6.30%		
	Basal-like	222	5.80%	1.075 (1.164, 1.409)	0.000
	Lost	1745			
Neoplasm Histologic Grade					
	1	181	4.70%		
	2	840	22%		
	3	1029	27%	1.281 (1.164, 1.409)	0.000
	Lost	1767			
Breast cancer type					
	1	979	25.60%		
	2	2316	60.70%		
	3	347	9.10%		
	4	122	3.20%	1.046 (0.986, 1.109)	0.134
Tumor stage					
	0	12	0.30%		
	1	695	18.20%		
	2	1524	39.90%		
	3	387	10.10%		
	4	30	0.80%	1.816 (1.652, 1.997)	0.000
	Lost	1169			
Primary Tumor Laterality					
	left	1551	40.60%		
	right	1423	37.30%	0.930 (0.831, 1.041)	0.210
	Lost	843			

ER, estrogen receptor; PR, progesterone receptor; HER2, human epidermal growth factor receptor 2; N-index, Nottingham prognostic index; HR, hazard ratio; CI, confidence interval.

### *SF3B1* mutation as a prognostic factor in luminal B and PR-negative breast cancer patients

**Figure 2:**
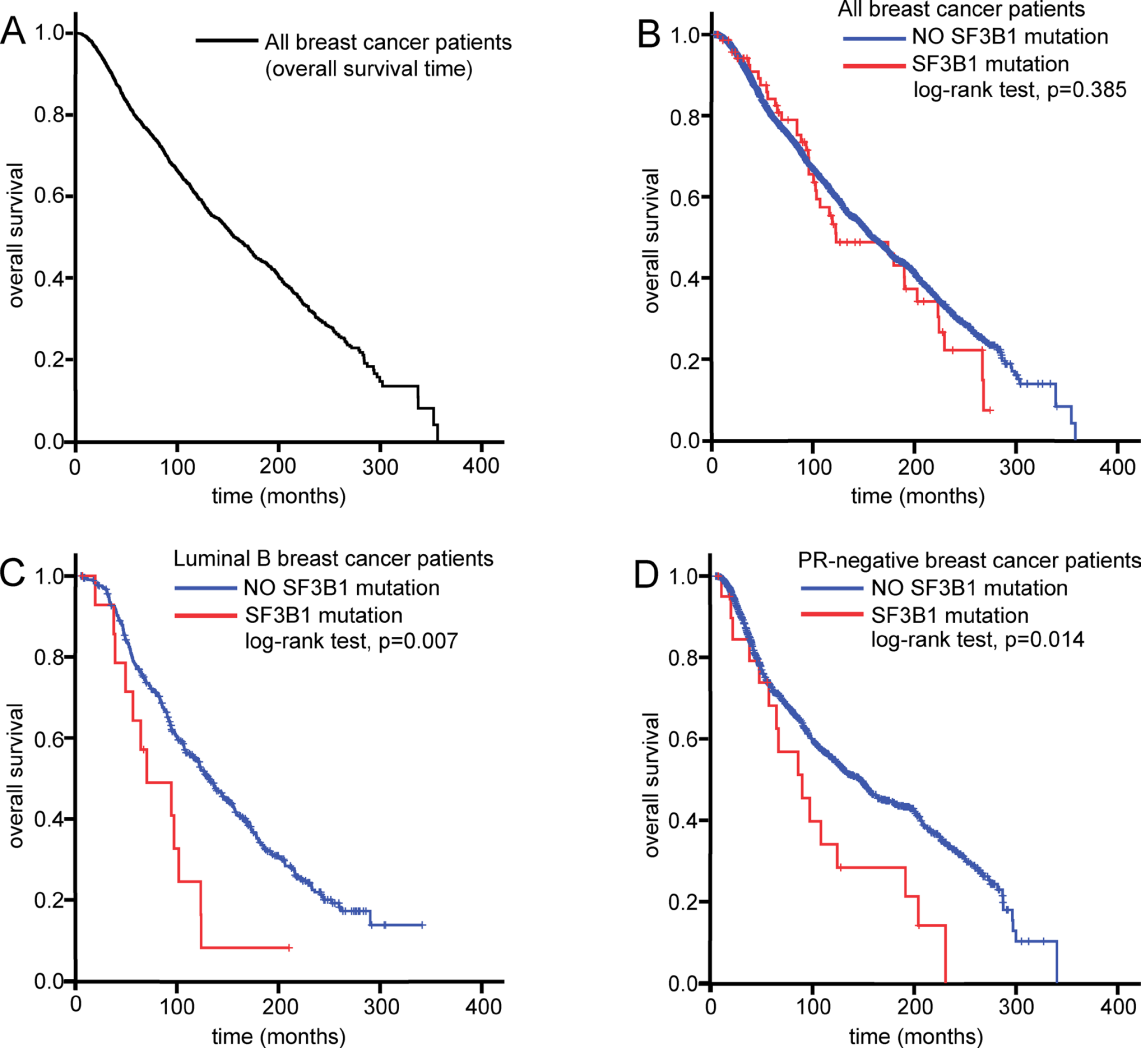
*SF3B1* mutation as prognostic factor in breast cancer patients Kaplan Meier curves showing overall survival in all breast cancer patients (**A**), as well as overall survival according to the presence of *SF3B1* mutations in all breast cancer patients (**B**), luminal B breast cancer patients (**C**), and progesterone receptor (PR)-negative breast cancer patients (**D**).

*SF3B1* mutation was not associated with OS in breast cancer patients; hence, subgroup analysis was performed to further investigate the clinical value of *SF3B1* mutations in these patients (Table 2). In the luminal B patient group, *SF3B1* mutation was significantly associated with the OS (hazard ratio [HR]: 2.188, 95% confidence interval [CI]: 1.225–3.907, *P* = 0.008). In the PR-negative group, the *SF3B1* mutation was also significantly associated with OS (HR: 1.845, 95% CI: 1.123–3.034, *P* = 0.016). Kaplan-Meier survival analysis showed that the *SF3B1* mutation was not an independent predictor for OS in breast cancer patients overall (log-rank test: *P* = 0.385). In the luminal B and PR-negative groups, however, the *SF3B1* mutation was a significantly independent prognostic factor for OS (log-rank test: *P* = 0.007 and *P* = 0.014, respectively) (Figure 2)

**Table 2:** *SF3B1* mutation as a prognostic factor for overall survival in all patient subgroups

all subgroup	HR (95% CI)	*P*
< 65 years	0.603 (0.250, 1.454)	0.260
≥ 65 years	1.005 (0.708, 1.426)	0.980
< 4.05 N-Index	1.112 (0.665, 1.859)	0.685
≥ 4.05 N-Index	1.271 (0.830, 1.946)	0.270
ER-positive	1.215 (0.875, 1.688)	0.245
ER-Negative	1.420 (0.199, 10.123)	0.726
PR-Positive	0.975 (0.637, 1.471)	0.905
PR-Negative	1.845 (1.123, 3.034)	0.016
HER2-Positive	1.103 (0.352, 3.454)	0.867
HER2-Negative	1.268 (0.905, 1.776)	0.168
Menopausal state pre-	0.049 (0.000, 15.553)	0.304
Menopausal state post-	1.231 (0.890, 1.072)	0.208
PAM50 and claudin-low subtype		
normal	1.915 (0.465, 7.885)	0.368
luminal A	0.876 (0.522, 1.469)	0.615
luminal B	2.188 (1.225, 3.907)	0.008
claudin-low	1.616 (0.397, 6.586)	0.503
HER2	1.443 (0.591, 3.525)	0.421
Basal-like	1.269 (0.177, 9.101)	0.813
Neoplasm Histologic Grade		
1	0.049 (0.000, 362.594)	0.506
2	1.049 (0.645, 1.704)	0.848
3	1.386 (0.866, 2.218)	0.173
Breast cancer type		
1	0.615 (0.152, 2.490)	0.495
2	1.209 (0.836, 1.749)	0.314
3	1.590 (0.584, 4.326)	0.364
4	1.166 (0.360, 3.770)	0.798
Tumor stage		
0	/	/
1	0.586 (0.188, 1.832)	0.358
2	1.297 (0.861, 1.954)	0.213
3	1.429 (0.353, 5.788)	0.617
4	/	/
Primary Tumor Laterality		
left	0.962 (0.800, 1.157)	0.680
right	1.012 (0.595, 1.720)	0.965

N-index, Nottingham prognostic index; ER, estrogen receptor; PR, progesterone receptor; HER2, human epidermal growth factor receptor 2; HR, hazard ratio; CI, confidence interval.

### Relationship between common prognostic factors and *SF3B1* mutation

Among 11 common prognostic factors investigated, univariate analysis showed that age, ER status, PR status, menopausal state, PAM50 and claudin-low subtype, and breast cancer type were significantly associated with *SF3B1* mutation in all breast cancer patients (*P* < 0.01) (Table 3). Age and ER status were significantly associated with *SF3B1* mutation on multivariate analysis (odds ratio: 1.037, 95% CI: 1.007–1.067, *P* = 0.015; and odds ratio: 6.055, 95% CI: 1.253–29.253, *P* = 0.025; respectively). Age and ER status were significantly associated with *SF3B1* mutation specifically in the luminal B and PR-negative subgroups as well (*P* < 0.02).

**Table 3:** Univariate Cox regression analysis of the association between *SF3B1* mutation and common prognostic factors

	All patients		Luminal B patients		PR-Negative patients	
	OR (95% CI)	*P*	OR (95% CI)	*P*	OR (95% CI)	*P*
Age	3.351 (2.093, 5.364)	0.000	6.414 (1.442, 28.528)	0.015	5.584 (2.187, 14.262)	0.000
N-Index	0.986 (0.581, 1.673)	0.958	1.455 (0.510, 4.154)	0.483	0.984 (0.385, 2.515)	0.972
ER status	7.920 (2.490, 25.192)	0.000	/	/	7.679 (2.271, 25.965)	0.001
PR	1.750 (1.069, 2.865)	0.026	/	/	/	/
HER2	0.458 (0.198, 1.063)	0.069	/	/	0.522 (0.153, 1.776)	0.298
Menopausal state	2.430 (1.159, 5.092)	0.019	1.880 (0.243, 14.522)	0.545	3.118 (0.725, 13.421)	0.127
PAM50 and claudin-low	0.807 (0.662, 0.982)	0.033	/	/	0.818 (0.615, 1.088)	0.168
Neoplasm Histologic Grade	0.859 (0.584, 1.263)	0.440	0.852 (0.333, 2.179)	0.738	0.507 (0.258, 0.994)	0.048
Breast cancer type	1.200 (1.017, 1.417)	0.031	1.643 (0.797, 3.384)	0.178	1.398 (0.709, 2.754)	0.333
Tumor stage	0.840 (0.575, 1.227)	0.367	0.985 (0.413, 2.350)	0.974	0.701 (0.335, 1.463)	0.344
Primary Tumor Laterality	1.030 (0.641, 1.655)	0.902	1.409 (0.466, 4.263)	0.543	1.006 (0.406, 2.493)	0.990

N-index, Nottingham prognostic index; ER, estrogen receptor; PR, progesterone receptor; HER2, human epidermal growth factor receptor 2; OR, odds ratio; CI, confidence interval.

### Predicting *SF3B1* mutation status using age at diagnosis and ER status

Receiver operating characteristic (ROC) curves were generated for all patients with breast cancer to identify the sensitivity and specificity of age at diagnosis and ER status in predicting *SF3B1* mutation. For age at diagnosis, the ROC curves showed a sensitivity of 65.8% and specificity 64.0%, with an area under the curve (AUC) of 0.639 (95% CI: 0.582–0.696, *P* < 0.000). For ER status, the ROC curves showed an AUC of 0.602 (95% CI: 0.548–0.656, *P* = 0.002). When age at diagnosis and ER status were assessed for their combined ability to predict *SF3B1* mutation, the ROC curve showed a higher sensitivity and specificity of 66.2% and 69.0%, respectively, with an AUC of 0.690 (95% CI: 0.639–0.740, *P* < 0.000).

**Figure 3:**
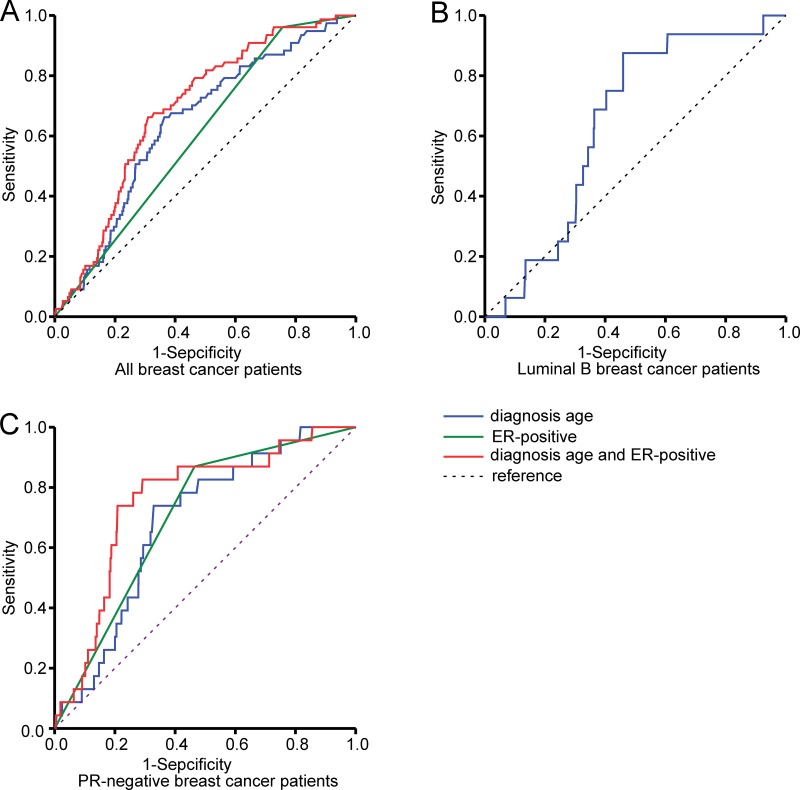
Receiver operating characteristic curves for predicting *SF3B1* mutation Shown are curves in all breast cancer patients (**A**), in luminal B breast cancer patients (**B**), and in progesterone receptor (PR)-negative breast cancer patients (**C**) according to age at diagnosis and estrogen receptor (ER) status.

For age at diagnosis in the luminal B group, ROC curves showed a sensitivity of 85.5% and specificity 54.1%, with an AUC of 0.643 (95% CI: 0.542–0.745, *P* = 0.051). In the PR-negative group, the ROC curve for age at diagnosis showed a sensitivity of 73.9% and specificity of 67.1%, with an AUC of 0.685 (95% CI: 0.597–0.773, *P* = 0.002). As for ER status, the ROC curve showed an AUC of 0.702 (95% CI: 0.612–0.793) (*P* = 0.001). When age at diagnosis and ER status were used in combination to predict *SF3B1* mutation, the ROC curve showed a higher sensitivity and specificity of 82.6% and 70.8%, respectively, with an AUC of 0.756 (95% CI: 0.664–0.848, *P* < 0.000) (Figure 3).

## DISCUSSION

SF3b is essential for pre-mRNA splicing, and mutations in its largest subunit (SF3B1/SF3b155) are linked to cancer [4, 16, 17]. The impact of *SF3B1* mutations on patient outcomes varies according to tumor type; for instance, it is associated with poor outcomes in CLL patients but with good prognoses in uveal melanoma patients [8, 11, 18–20]. Human breast cancers are heterogeneous; Blows et al. showed that ER-positive breast cancer patients had varied outcomes and responses to therapy [21]. A study by Nik-Zainal et al. in which whole genomes from 560 breast cancers and non-neoplastic tissue were sequenced revealed 3479652 somatic base substitutions, 371993 small indels, and 77695 rearrangements [22]. The high number of gene mutations and diversity of genomic drivers may explain this disease’s clinical heterogeneity. An *SF3B1* mutation can cause abnormal pre-RNA splicing that can lead to tumorigenesis, tumor drug resistance, or others detrimental features [20, 23, 24]. Therefore, *SF3B1* mutation may be useful as a prognostic indicator in different tumors.

A study by Pereira et al. showed that mutations in driver genes were associated with the prognosis of breast cancer patients. For instance, *MAP3K1* and *GATA3* mutations were associated with longer survival, and *TP53* mutations with shorter survival, in ER-positive patients but not in ER-negative patients. Conversely, *PIK3CA* mutations were associated with shorter survival in ER-negative patients, but not in ER-positive patients. Their study also showed associations between mutations in driver genes and clinicopathological parameters; for example, mutations in *PIK3CA*, *GATA3*, *KMT2C*, and *CBFB* were associated with lower grade tumors in ER-positive patients, while *TP53* mutations were associated with higher grade tumors. *GATA3*, *CBFB*, *CDH1*, *KMT2C*, and *SF3B1* mutations were also associated with age at diagnosis [14].

In our study, we further analyzed the clinic value of *SF3B1* mutations in breast cancer patents. *SF3B1* mutations were not significantly associated with survival outcome in breast cancer patients overall. However, these mutations were associated with worse outcomes in PR-negative and luminal B patients. In the PR-negative patient subgroup, *SF3B1* mutations were associated with age at diagnosis, ER status, and histologic grade; in the luminal B subgroup, *SF3B1* mutations were associated only with the age at diagnosis. The multivariate logistic regression model revealed that *SF3B1* mutations were associated with age at diagnosis and ER status in all patients as well as in the PR-negative subgroup.

Because *SF3B1* mutations were associated with worse outcomes in the PR-negative and luminal B subgroups, we analyzed whether these mutations were significantly associated with the age at diagnosis and ER status with the hypothesis that the age and ER status can predicting the existence of an *SF3B1* mutation. We found that, when age at diagnosis and ER status were assessed in combination, the prediction of *SF3B1* mutations had a slightly higher sensitivity, specificity, and AUC than the age at diagnosis in all patients. In the PR-negative subgroup, age and ER combined had a higher sensitivity (82.6%), specificity (70.8%), and AUC (0.756) in terms of predicting *SF3B1* mutations than age alone.

Maguire et al. showed that *SF3B1* K700E mutations are associated with differential gene splicing in breast cancer, including of *TMEM14C*, *RPL31*, *CRNDE*, *DYNLL1*, *ICA1*, *RPL24*, and *MTERFD3*. Cell lines carrying the *SF3B1* mutation were sensitive to the SF3b complex inhibitor spliceostatin A, which suppressed tumor growth. Hence, the spliceosome SF3b complex may be a potential therapeutic target [11, 25].

In conclusion, our analysis of TCGA revealed that *SF3B1* mutations are frequently found in breast cancer patients, and that they are poor prognostic indicators in PR-negative and luminal B breast cancer patients. *SF3B1* mutations were found to be significantly associated with the age at diagnosis and/or ER status in PR-negative and luminal B breast cancer patients. Moreover, combining the age at diagnosis and ER status could better predict the existence of *SF3B1* mutations. As demonstrated by spliceostatin A, the SF3b complex may be a novel therapeutic target for breast cancer patients with *SF3B1* mutations.

## MATERIALS AND METHODS

### Analysis using the cancer genome atlas (tcga) database

We analyzed SF3B1 mutation data and clinic data of breast cancer patients from TCGA database (www.cbioportal.org/). We enrolled 2059, 1105, 103, and 100 invasive breast carcinoma patients from the METABRIC, TCGA (provisional), broad, and Sanger datasets, respectively, in our study. Our institutional review board approved this study, which was performed according to the principles of the Declaration of Helsinki. There were 81 patients with *SF3B1* mutations among 3817 patients with invasive breast carcinoma. Breast cancers were divided into four types: invasive breast carcinoma (979), breast invasive ductal carcinoma (2316), breast invasive lobular carcinoma (347), and breast mixed ductal and lobular carcinoma (122). Fifty-three patients were not classified because of the rarity of their tumor types, including adenoid cystic breast cancer, phyllodes tumor of the breast, and unclassified breast cancer.

### Risk factor analysis

The following parameters were investigated for their role in the prognosis of patients with breast cancer: age at diagnosis, N-index, ER status, PR status, HER2 status, menopausal state, PAM50 and claudin-low subtype, histological grade, breast cancer type, tumor stage, and primary tumor laterality. Additionally, *SF3B1* was also analyzed as a prognostic marker. We divided the age and N-Index into dichotomous variables (< 65 vs. ≥ 65 years and < 4.05 vs. ≥ 4.05, respectively); these cutoff values were based on OS.

### Statistical analysis

All dichotomous variables were analyzed by using a Cox regression models in all patients. Next, we divided all dichotomous prognostic factors into two subgroups in which the *SF3B1* mutation as independent prognostic factor was evaluated. The Kaplan-Meier method was used to estimate survival function of each variable that was found to be a significant factor. Univariate analysis was performed to identify significant independent prognostic factors for OS. The Cox proportional hazards model was used to estimate the HRs and CIs of potential prognostic factors in all patients, including that of *SF3B1* mutation in the aforementioned subgroups. Patients who were positive for significant prognostic factors were subjected to Kaplan-Meier analysis to clarify the role of the *SF3B1* mutation on their survival. A logistic regression model was used to analyze associations between the investigated prognostic factors and *SF3B1* mutation. Variables identified via univariate analysis (α = 0.25) were subjected to multivariable logistic regression analysis to determine their association with *SF3B1* mutations. ROC curves were constructed to predict the sensitivity and specificity of SF3B1 mutations according to the AUC and 95% CI. We also assessed the combined effects of age at diagnosis and ER status on the occurrence of *SF3B1* mutations. All analyses were performed using SPSS for Windows, version 20.0 (SPSS, Chicago, IL, USA).

## Abbreviations

AUC: area under the curve
CI: confidence interval
CLL: chronic lymphocytic leukemia
ER: estrogen receptor
HR: hazard ratio
HER2: human epidermal growth factor receptor 2
N-index: Nottingham prognostic index
OR: odds ratio
OS: overall survival
PR: progesterone receptor
ROC: receiver operating characteristic
TCGA: The Cancer Genome Atlas
